# The 2025 paradigm shift in urothelial carcinoma pharmacotherapy: from chemotherapy to ADCs and ICIs

**DOI:** 10.3389/fcell.2026.1904484

**Published:** 2026-07-10

**Authors:** Yong Liang, Duoji Zhaxi, Meng Fu, Junjie Hu

**Affiliations:** 1 Department of Urology, Lhasa People’s Hospital, Lhasa, Xizang, China; 2 Department of Urology, Beijing Tsinghua Changgung Hospital, School of Clinical Medicine, Tsinghua University, Beijing, China

**Keywords:** antibody-drug conjugate, bladder cancer, immune checkpoint inhibitor, upper tract urothelial carcinoma, urothelial carcinoma

## Abstract

The pharmacotherapy of urothelial carcinoma (UC) has undergone a paradigm shift from conventional chemotherapy and BCG to novel agent-based strategies, including immune checkpoint inhibitors (ICIs) and antibody-drug conjugates (ADCs), moving from later-line to frontline, monotherapy to combination, and systemic to locoregional-systemic synergy. In non-muscle-invasive bladder cancer, ICI-BCG combinations and ADC-BCG regimens improve treatment responses and patient outcomes, while the intravesical delivery system TAR-200 also shows therapeutic potential. For muscle-invasive bladder cancer, ADC/ICI-based perioperative strategies alongside biomarker-guided approaches have shown significantly improved survival. In upper tract urothelial carcinoma, ADC/ICI-based combinations show promise in neoadjuvant, kidney-sparing, and adjuvant settings. For advanced or metastatic UC, first-line enfortumab vedotin plus pembrolizumab demonstrates superior survival over chemotherapy, and various clinical trials of novel agent-based strategies are ongoing. Challenges include optimizing patient selection, determining ideal treatment duration, managing treatment-related adverse events, and preserving organ function. Future directions emphasize precision-oriented, function-preserving, and biomarker-driven individualized management.

## Introduction

Urothelial carcinoma (UC) is one of the most common malignancies worldwide and is typically classified based on depth of tumor invasion and anatomical site into non-muscle-invasive bladder cancer (NMIBC), muscle-invasive bladder cancer (MIBC), and upper tract urothelial carcinoma (UTUC), with bladder cancer (BCa) accounting for 90%–95% of cases and UTUC for only 5%–10% (Masson-Lecomte et al., 2025; Siegel et al., 2025). Treatment strategies for UC vary by stage and location, with the core principle being standardized surgical intervention combined with systemic therapy (van der Heijden et al., 2025); for patients with advanced or unresectable disease, the development of drug therapies has been particularly rapid. For a long time, platinum-based chemotherapy was the standard treatment for advanced UC, yielding a median overall survival (OS) of approximately 12.7–15.8 months (Bellmunt et al., 2012); however, about 30%–50% of patients are ineligible for cisplatin due to severe adverse effects, particularly renal impairment (Galsky et al., 2011; Dash et al., 2006). Meanwhile, UC cells can acquire cisplatin resistance through multiple pathways, including the acquisition of tumor stemness, metabolic and immune reprogramming of the tumor microenvironment, enhancement of DNA repair capacity, epigenetic modulation, and suppression of apoptosis (Ma et al., 2026). In recent years, novel agents such as immune checkpoint inhibitors (ICIs) and antibody-drug conjugates (ADCs) have profoundly reshaped the UC treatment landscape. ICIs release the brakes on the immune system, reactivating T cells to respond to tumor antigens and eradicate tumor cells (Li et al., 2026). High tumor mutational burden (TMB) is often associated with greater neoantigen load, suggesting potential sensitivity to immunotherapy (Sun et al., 2025). UC is characterized by a relatively high TMB and neoantigen load, suggesting potential responsiveness to immunotherapy (Yarchoan et al., 2017; Alexandrov et al., 2013; Tn and Rd, 2015). ADCs, which conjugate cytotoxic agents to antibodies to enable precise drug delivery to tumor cells, have demonstrated potential for superior efficacy and safety compared with traditional chemotherapy (Chen B. et al., 2025). HER2 is an important oncogenic molecule that regulates cell proliferation and invasion, correlating with aggressive clinicopathological features in UC (Luo et al., 2026; Alibeiginejad et al., 2026); Nectin-4 is a tumor-associated antigen that is highly expressed on the surface of the majority of UC cells, while its expression is restricted in non-neoplastic urothelium (Kobayashi et al., 2025-06). As the efficacy of ADC is based on the expression level of its targeted antigen on tumor cells, the high expression of Nectin-4 in UC cells provides a foundation for the efficacy of the ADC agent enfortumab vedotin (EV) (Kobayashi et al., 2025-06). HER2 is less frequently expressed than nectin-4 in UC; thus, the HER2-targeting ADC disitamab vedotin (RC48) may require individualized use based on tumor HER2 expression (Fan et al., 2022; Liu Z. et al., 2025).

Several pivotal clinical studies have driven the evolution of treatment strategies for UC. From investigations of different therapeutic regimens in advanced UC, such as KEYNOTE-045/052 (Balar et al., 2023), JAVELIN Bladder 100 (Sridhar et al., 2024), and TROPiCS-04 (Powles et al., 2025a), to the breakthrough achieved in the EV-302 (Powles TB. et al., 2025) study, novel agents are progressively moving from later-line settings to first-line and maintenance therapy. Currently, pembrolizumab plus EV has become the new standard first-line regimen for locally advanced or metastatic UC (la/mUC) (Powles TB. et al., 2025). And concurrently, the application scenarios for novel drugs are extending from advanced to perioperative and even early-stage UC, which remain under clinical evaluation. Studies including IMvigor010 (Bellmunt et al., 2021), PURE-01 (Basile et al., 2022), and NCT03924895 (Vulsteke et al., 2026-02) have explored the use of novel drug in perioperative UC management, while trials such as CREST (Shore et al., 2025), ALBAN (Roupret et al., 2026), NCT06187506 (Shen et al., 2025), and Formula-01 (Tan et al., 2025) are further investigating their role in early-stage disease like NMIBC. Novel localized drug delivery systems exemplified by TAR-200 have expanded their application from NMIBC to MIBC, yielding encouraging progress (Necchi et al., 2025a). Additionally, emerging therapeutic modalities, including individualized vaccines, are also undergoing preliminary exploration in UC (Saxena et al., 2025). In recent years, multiple clinical trials in UC have reported updated results, demonstrating exciting advances that are poised to drive significant changes in UC treatment guidelines and clinical practice (Table 1). This review aims to systematically summarize key clinical research progress in UC pharmacotherapy in 2025, providing a high level of evidence-based medical instruction for future UC management.

**TABLE 1:** Key clinical trials of urothelial carcinoma.

Trial, phase, random or not (registration number)	Disease stage	Treatment (experimental arm vs. control arm)	Sample size	Key efficacy outcomes	Adverse events	Refs
CREST, phase 3, random (NCT04165317)	NMIBC	Sasanlimab + BCG-I + M (arm A) vs. Sasanlimab + BCG-I (arm B) vs. BCG-I + M (arm C)	352 vs. 352 vs. 351	36-month EFS rate 82.1% and 74.8% (arm A vs. arm C)	≥3 grade TRAEs 29.1% (arm A) vs. 6.3% (arm C)	Shore et al. (2025)
ALBAN, phase 3, random (NCT03799835)	NMIBC	Atezolizumab + BCG-I + M vs. BCG-I + M	262 vs. 255	HR of EFS (0.98, P = 0.91)	≥3 grade TRAEs 22.7% (exp.) vs. 8.8% (contr.)	Roupret et al. (2026)
POTOMAC, phase 3, random (NCT03528694)	NMIBC	Durvalumab + BCG-I + M (arm A) vs. Durvalumab + BCG-I (arm B) vs. BCG-I + M (arm C)	339 vs. 339 vs. 340	HR of DFS (0.68, p = 0.015)	Grade 3–4 TRAEs 21% (arm A) vs. 4% (arm C)	De Santis et al. (2025)
NCT06187506, phase 2, not random	NMIBC	RC48 + BCG	20	For residual tumor or CIS: 11 pts (100% cCR at 3 months), 5 pts (100% cCR at 6 months) For complete tumor resection: 3 pts (100% 6-month EFS rate)	TRAEs 65%; Grade 3 TRAEs 10%	Shen et al. (2025)
Formula-01, phase 2, not random (ChiCTR2400080767)	NMIBC	RC48 + BCG	78	1-year DFS 92.0%	Grade 3–4 TRAEs 12.8%	Tan et al. (2025)
NCT06378242, phase 1, not random	NMIBC	Intravesical RC48	9	6‐months RFS rates 100% (8/8), 12‐months RFS rates 87.5% (7/8), 6‐month and 12-month PFS rates 100% (8/8)	≥3 grade TRAEs 0%	Chen et al. (2020)
SunRISe-1, phase 2b, random (NCT04640623)	NMIBC	TAR-200 + cetrelimab (arm A) vs. TAR-200 monotherapy (arm B) vs. cetrelimab monotherapy (arm C) in BCG-unresponsive CIS pts, and vs. TAR-200 monotherapy in BCG-unresponsive high-risk papillary disease-only NMIBC pts (arm D)	53 vs. 85 vs. 28 vs. 52	arm B: CR rate 82.4%, median duration of response 25.8 months	≥3 grade TRAEs 12.9%	Daneshmand et al. (2025)
KEYNOTE-905/EV303, phase 3, random (NCT03924895)	MIBC	Perioperative EV + pembrolizumab vs. surgery alone	170 vs. 174	pCR rate 57.1% vs. 8.6% HR of EFS (0.4, P < 0.001) HR of OS (HR = 0.5, P < 0.001)	≥3 grade TRAEs 45.5% (exp.)	(Vulsteke et al., 2026-02)
RC48-C017, phase 2, not random (NCT05297552)	MIBC	Neoadjuvant RC48 + perioperative toripalimab	47	12-month EFS rate 91.0% 18-month EFS rate 81.5% 12-month OS rate 95.7% 24-month OS rate 91.3%	No new safety signals	Sheng et al. (2026)
RETAIN-2, phase 2, not random (NCT04506554)	MIBC	Neoadjuvant AMVAC + nivolumab	80	pT0 rate 46%, <T2 rate 63% in RC group metastasis-free bladder-preservation rate 78% pts in AS group	grade 3–4 TRAEs 19%	Ghatalia et al. (2025)
SunRISe-4, phase 2, random (NCT04919512)	MIBC	Neoadjuvant TAR-200 + cetrelimab vs. neoadjuvant cetrelimab	101 vs. 58	pCR rate (38% vs. 28%) pOR rate (53% vs. 44%) 1-year RFS rate (77% vs. 64%)	≥3 grade TRAEs (15.8% vs. 10.3%)	Necchi et al. (2025b)
CheckMate 274, phase 3, random (NCT02632409)	MIUC	Adjuvant nivolumab vs. placebo	353 vs. 356	HR of DFS (0.74, 95% CI 0.61–0.90) in all pts	No new safety signals	Galsky et al. (2026)
IMvigor011, phase 3, random (NCT04660344)	MIBC	Adjuvant atezolizumab vs. placebo in ctDNA-positive pts	167 vs. 83	In ctDNA-positive pts HR of DFS (0.64, 95% CI 0.47–0.87) HR of OS (0.59, 95% CI 0.39–0.90) In ctDNA-negative pts 1-year DFS 95% 2-year DFS 88%	grade 3–4 TRAEs (7% vs. 4%) grade 5 TRAEs (2% vs. 0%)	Powles et al. (2025c)
WUTSUP-01, phase 2, not random (ChiCTR2500102886)	UTUC	Neoadjuvant toripalimab + gemcitabine and cisplatin	49	In pts underwent RNU, pCR rate 30.2%, ORR rate 76.7% 2-year PFS rate 80.6% 2-year OS rate 93.5%	Grade 3–4 TRAEs 37%	Chen et al. (2025b)
iNDUCT-GETUG V08, phase 2, not random (NCT04617756)	UTUC	Neoadjuvant Durvalumab + gemcitabine + cisplatin (arm A) or carboplatin (arm B)	31 vs. 19	pT0 rate (13% vs. 5%) pTa/pT1 rate (30% vs. 42%)	≥3 grade TRAEs 48%	Houédé et al. (2025)
WUTSUP-03, phase 2, not random (ChiCTR2600121637)	UTUC	Endoscopic thulium laser ablation + RC48 + ICI(toripalimab or tislelizumab)	33	1-year local RFS rate 67% 2-year local RFS rate 64% 2-year CFS rate 94%	1-year ureteral stricture rate 12% >3 grade TRAEs 0%	Ye et al. (2025)
NCT05837806, phase 2, not random	UTUC	Neoadjuvant tislelizumab + RC48	15	cCR rate 25% no death or recurrence reported in the median follow-up 117 days	grade 3 TRAEs 10.5%	Chen et al. (2025c)
REBACARE, phase 2, not random (EudraCT number 2017-000949-53)	UTUC	Intravesical chemotherapy instillation before radical surgery	178	For pts without d-URS HR of IVR (0.33, 95% CI 0.12–0.87)	>2 grade toxicity 0%	van Doeveren et al. (2025)
NCT05917158, phase 2, not random	UTUC	Adjuvant RC48 + toripalimab	45	1-year DFS rate 90.0%	≥3 grade TRAEs 20%	Liu et al. (2025b)
EV-302/KEYNOTE-A39, phase 3, random (NCT04223856)	la/mUC	EV + pembrolizumab vs. chemotherapy	442 vs. 444	HR of PFS (0.48, 95% CI 0.41–0.57) HR of OS (0.51, 95% CI 0.43–0.61)	≥3 grade TRAEs (57.3 vs. 69.5)	Powles et al. (2025b)
RC48-C016, phase 3, random (NCT05302284)	la/mUC	RC48 + toripalimab vs. chemotherapy	243 vs. 241	HR of PFS (0.36, P < 0.001) HR of OS (0.54, P < 0.001) ORR 76.1% vs. 50.2%	≥3 grade TRAEs (55.1% vs. 86.9%)	Sheng et al. (2025)
DISCUS, phase 2, random (NCT06892860)	a/mUC	3 vs. 6 cycles (3C arm versus 6C arm) of chemotherapy followed by avelumab	133 vs. 134	HR of PFS (1.05, P = 0.79) HR of OS (1.15, P = 0.56) ORR 24% vs. 27% QoL change favouring 3C (+8.5 points, P = 0.016)	Grade 3–4 TRAEs (40% vs. 54%)	Powles et al. (2026)
JAVELIN Bladder Medley, phase 2, random (NCT05327530)	la/mUC	avelumab + SG maintenance vs. avelumab maintenance	74 vs. 37	HR of PFS (0.49, 95% CI 0.31–0.76)	≥3 grade TRAEs (69.9% vs. 0%)	Hoffman-Censits et al. (2025)

Abbreviations: NMIBC, non-muscle invasive bladder cancer; MIBC, muscle invasive bladder cancer; MIUC, muscle invasive urothelial carcinoma; UTUC, upper tract urothelial carcinoma; la/mUC, locally advanced or metastatic urothelial carcinoma; a/mUC, advanced or metastatic urothelial carcinoma; CIS, carcinoma *in situ*; mo, month; yr, year; EFS, event-free survival; DFS, disease-free survival; RFS, recurrence-free survival; PFS, progression-free survival; OS, overall survival; CFS, conversion-free survival; IVR, intravesical recurrence; ORR, objective response rate; pCR, pathological complete response; cCR, clinical complete response; HR, hazard ratio; CI, confidence interval; QoL, quality of life; pts, patients; BCG-I + M, *bacillus* calmette-guérin induction + maintenance; BCG-I, *bacillus* calmette-guérin induction; TRAEs, treatment-related adverse events; EV, enfortumab vedotin; RC48, Disitamab vedotin; ICI, immune checkpoint inhibitor; AMVAC, accelerated methotrexate, vinblastine, doxorubicin, and cisplatin; SG, sacituzumab govitecan.

## Non-muscle-invasive bladder cancer

NMIBC accounts for approximately 75% of BCa cases, with transurethral resection of bladder tumor (TURBT) as the primary treatment, followed by risk-stratified adjuvant therapy such as intravesical BCG (Gontero et al., 2024). High-risk and very-high-risk NMIBC patients face substantial progression risks, with 10-year progression rates of 14% and >50%, respectively (Gontero et al., 2024); among high-grade NMIBC patients, the 10-year recurrence rate reaches 74% (Barton, 2013). Although BCG induction plus maintenance (BCG-I/M) is the standard treatment for high-risk NMIBC, many patients still experience recurrence and progression, alongside challenges such as severe BCG adverse reactions and BCG shortages (Oddens et al., 2013; Tang et al., 2026). Radical cystectomy (RC) offers reliable tumor control but its postoperative complications severely impact quality of life and can be life-threatening (Raj et al., 2007; Willis et al., 2015; Tempo et al., 2025). Recent advances in novel drug development, innovative delivery routes, and multi-agent combinations have expanded options beyond conventional BCG therapy or RC for high-risk NMIBC.

Intravesical BCG could upregulate PD-L1 expression in tumor tissues, creating a favorable microenvironment for ICIs (Maas et al., 2024). Early studies like KEYNOTE-057 in 2021 demonstrated that ICIs such as pembrolizumab can improve outcomes in BCG-unresponsive NMIBC (Balar et al., 2021). By 2025, substantial clinical evidence has accumulated on novel immunotherapies combined with BCG in high-risk NMIBC. Three randomized phase III trials (each with several hundred patients per arm) including CREST (Shore et al., 2025), ALBAN (Roupret et al., 2026), and POTOMAC (De Santis et al., 2025), evaluated different ICIs plus BCG-I/M versus BCG-I/M alone for BCG-naive high-risk NMIBC patients. Although no mature overall survival (OS) data are available, sasanlimab (Shore et al., 2025) and durvalumab (De Santis et al., 2025) regimens showed significant advantages in event-free survival (EFS) or disease-free survival (DFS) over BCG monotherapy. These three studies collectively demonstrate the potential benefit of ICI-BCG combinations in NMIBC from different drug perspectives. However, all three also reported notably higher rates of grade 3–4 treatment-related adverse events (TRAEs) in the ICI + BCG arms, indicating that the balance between survival benefit and safety requires further investigation (Shore et al., 2025; Roupret et al., 2026; De Santis et al., 2025). The efficacy of ADC combined with BCG in high- or very-high-risk NMIBC has also been preliminarily validated. Multiple studies have reported on the RC48 plus BCG. In very-high-risk NMIBC patients with HER2 expression (IHC 1+/2+/3+) who declined or were unfit for RC, Shen et al. (Shen et al., 2025) conducted a prospective, open label, single-center study for BCG-naive patients, reporting that RC48 plus BCG achieved 3-month and 6-month clinical complete response (cCR) rates of 100% in patients with residual tumor or carcinoma *in situ* (CIS) (n = 15, 11 and 5 patients respectively for each time point), and a 6-month EFS rate of 100% in three of five patients who underwent complete tumor resection. The overall TRAE rate was 65%, with grade 3 TRAEs accounting for 10% (Shen et al., 2025). In another non-randomized phase II trial of 78 patients with HER2-overexpressing (IHC 2+/3+) high-risk NMIBC, Tan et al. (Tan et al., 2025) reported a 1-year DFS rate of 92% with RC48 plus BCG, and a grade 3–4 TRAE rate of 12.8%. To reduce systemic toxicity of intravenous ADCs, a phase I trial (NCT06378242) of intravesical RC48 for HER2-positive high-risk NMIBC is ongoing, with preliminary results showing good tolerability and initial efficacy (Chen et al., 2020). These studies suggest that HER2-targeting ADC show promising efficacy in NMIBC, and future RCT results are awaited. Regarding novel intravesical delivery systems, the phase IIb SunRISe-1 study reported that in BCG-unresponsive CIS NMIBC patients, TAR-200 monotherapy achieved a complete response rate of 82.4% and a median response duration of 25.8 months, outperforming TAR-200 plus cetrelimab or cetrelimab alone (Daneshmand et al., 2025). These findings importantly complement conventional intravesical BCG therapy, demonstrate promising prognostic improvements, and provide high-quality evidence for optimizing bladder-preserving strategies in high-risk/very-high-risk NMIBC.

## Muscle-invasive bladder cancer

In the field of MIBC, RC has long been the gold standard for local treatment of non-metastatic MIBC (van der Heijden et al., 2025). The SWOG-8710 study in 2003 established the role of neoadjuvant methotrexate, vinblastine, doxorubicin, and cisplatin (MVAC) chemotherapy in MIBC management (Grossman et al., 2003), and the VESPER study in 2022 advanced the preferential position of ddMVAC in neoadjuvant therapy (Pfister et al., 2022). The 2024 NIAGARA study demonstrated that perioperative Durvalumab immunotherapy combined with neoadjuvant chemotherapy (NAC) confers survival benefits without increasing TRAEs, preliminarily establishing the role of immunotherapy in MIBC perioperative management (Powles et al., 2024). In 2025, novel therapeutic regimens based on ICIs and/or ADCs are profoundly reshaping the perioperative treatment landscape of MIBC through a series of clinical trial results.

In the KEYNOTE-905/EV303 randomized phase III trial for cisplatin-ineligible MIBC perioperative therapy, pembrolizumab combined with the Nectin-4-targeting ADC EV significantly improved the pathological complete response (pCR) rate (57.1% vs. 8.6%) and enhanced EFS (HR = 0.4, P < 0.001) and OS (HR = 0.5, P < 0.001) (Vulsteke et al., 2026-02). Although all patients in the combination arm experienced adverse events, with higher rates of grade ≥3 TRAEs (45.5% vs. 0%) and drug-related discontinuation of trial drug (37.1% vs. 0%), only 2 of the 13 deaths in this arm were drug-related, and the overall safety profile was consistent with previous studies, with no new safety signals identified (Vulsteke et al., 2026-02). Updated results from a single-arm phase II trial (NCT05297552) evaluating perioperative toripalimab combined with neoadjuvant RC48 in untreated MIBC patients showed that, at a median follow-up of 26.4 months, the 12-month and 18-month EFS rates were 93.2% and 80.9%, respectively, in the 33 operated patients, and 91.0% and 81.5% in the overall population; the 12-month and 24-month OS rates were 95.7% and 91.3%, respectively, with manageable TRAEs (Sheng et al., 2026). These two studies show the efficacy of ADC plus immunotherapy in perioperative MIBC treatment and are promising as a potential first-line option for cisplatin-tolerant MIBC. In the phase II RETAIN-2 study, 71 MIBC patients received neoadjuvant accelerated methotrexate, vinblastine, doxorubicin, and cisplatin (AMVAC) combined with nivolumab, with pre-treatment TURBT specimens tested for ATM, ERCC2, or RB1 mutations (Ghatalia et al., 2025). Those harboring >1 mutation and achieving cCR after neoadjuvant therapy proceeded to active surveillance (AS), while others received intravesical therapy, chemoradiotherapy, or RC. Preliminary results showed a pT0 rate of 46% in the RC group, and at a median follow-up of 18.4 months, 78% of the AS group remained metastasis-free with bladder preservation (Ghatalia et al., 2025). This study highlights the marked efficacy of neoadjuvant chemo-immunotherapy and suggests that molecular biomarkers facilitate refined management of MIBC patients, with potential to select bladder-preserving candidates after neoadjuvant chemo-immunotherapy. In local treatment exploration, TAR-200 is gradually being explored in MIBC. The SunRISe-4 study evaluated the neoadjuvant efficacy of TAR-200 combined with intravenous cetrelimab in cisplatin-ineligible or cisplatin-refusing MIBC patients (Necchi et al., 2025b). Preliminary results showed that the combination arm outperformed cetrelimab monotherapy in pCR rate (38% vs. 28%), objective response rate (ORR, ≤ypT1N0; 53% vs. 44%), and 1-year recurrence-free survival (RFS; 77% vs. 64%), with no new safety signals observed (Necchi et al., 2025b). Moreover, preoperative urinary tumor DNA (utDNA) status was significantly associated with pCR, and preoperative circulating tumor DNA (ctDNA) status was significantly associated with RFS in this study (Necchi et al., 2025b).

In the adjuvant setting, the U.S. Food and Drug Administration has approved nivolumab for high-risk recurrent UC patients after surgery (van der Heijden et al., 2025). While the prior randomised phase III IMvigor010 study found no DFS benefit from adjuvant atezolizumab in MIUC patients (Bellmunt et al., 2021); in contrast, the recent results from the phase III randomized, double-blind, multicenter CheckMate 274 trial showed that adjuvant nivolumab effectively improves DFS in high-risk MIUC patients, with postoperative ctDNA positivity (approximately 40% of patients) being an important factor influencing efficacy (Galsky et al., 2026). Regarding whether ctDNA can guide MIBC perioperative therapy, the IMvigor011 study randomized MIBC patients with postoperative ctDNA positivity to receive adjuvant atezolizumab, demonstrating significantly prolonged DFS (9.9 months vs. 4.8 months) and OS (32.8 months vs. 21.1 months), while untreated patients with persistently negative postoperative ctDNA achieved a 2-year DFS of 88% (Powles et al., 2025c). Additionally, the NIAGARA study on the efficacy of perioperative durvalumab + NAC found that clearance of perioperative utDNA was significantly associated with longer EFS (HR 0.24), with a pCR rate of 72% in preoperative utDNA-negative patients versus 18% in positive patients; the 24-month EFS rate was only 55% in patients positive for both preoperative utDNA and ctDNA, but as high as 90% in double-negative patients (Van Der Heijden et al., 2026). These clinical trials suggest that risk stratification based on liquid biopsy and other molecular biomarkers could serve as a key indicator of MIBC perioperative treatment prognosis and hold promise as an important reference for refining MIBC perioperative management.

## Upper tract urothelial carcinoma and advanced or metastatic urothelial carcinoma

UTUC, a relatively rare subtype of UC, has research evidence that lags far behind that of BCa. Due to heterogeneity in both anatomical location and genetic alterations, the disease management strategies and treatment responses for UTUC exhibit certain differences from those for BCa (Sfakianos et al., 2021; Soria et al., 2017). Radical nephroureterectomy (RNU) is the primary curative treatment for localized UTUC, and the randomized phase III POUT trial established the role of platinum-based adjuvant chemotherapy following RNU (Birtle et al., 2024). However, renal parenchyma loss due to RNU compromises postoperative renal function reserve, limiting the delivery and efficacy of adjuvant chemotherapy; moreover, the difficulty of obtaining accurate clinical and pathological features preoperatively restricts the use of neoadjuvant therapy in UTUC (Soria et al., 2017; Birtle et al., 2024). Thus, optimizing perioperative treatment strategies for UTUC, including neoadjuvant therapy before RNU, kidney-sparing approaches, and adjuvant therapy after radical surgery, is particularly important. In the neoadjuvant setting, the single-arm phase II WUTSUP-01 study evaluated the efficacy and safety of toripalimab combined with NAC (cisplatin and gemcitabine) before RNU in patients with locally advanced UTUC (Chen K. et al., 2025). Updated data showed that among 43 patients who underwent RNU, the pCR rate was 30.2%, the ORR was 76.7%, the 2-year PFS rate was 80.6%, the 2-year OS rate was 93.5%, and the incidence of grade 3–4 TRAEs was 37%, primarily chemotherapy-induced myelosuppression (Chen K. et al., 2025). Another study, phase II iNDUCT-GETUG V08 trial, evaluated neoadjuvant durvalumab plus gemcitabine and cisplatin or carboplatin in high-risk UTUC, demonstrating favorable tumor downstaging and a manageable safety profile, with no severe immunotherapy-mediated toxicity was observed (Houédé et al., 2025). These two studies reflect attempts to combine chemotherapy with immunotherapy in the neoadjuvant treatment of UTUC. For neoadjuvant ADC plus immunotherapy before surgery in UTUC, a single-arm phase II study (NCT05837806) assessed RC48 combined with tislelizumab in patients with HER2 expression (1+/2+/3+), with preliminary results showing a cCR rate of 25% (3/12) and acceptable safety (Chen H. et al., 2025). The ADC-immunotherapy strategy also performed well in kidney-sparing treatment for locally advanced high-risk UTUC. Endoscopic thulium laser ablation combined with RC48 and an ICI (toripalimab or tislelizumab) achieved a high kidney preservation rate, with a 2-year conversion-free survival rate of 94% (Ye et al., 2025). Beyond systemic therapy, preoperative intravesical instillation chemotherapy in the single-arm phase II REBACARE study for UTUC was found to reduce intravesical recurrence risk in patients who did not undergo diagnostic ureteroscopy, suggesting the potential of endoluminal therapy in the neoadjuvant setting (van Doeveren et al., 2025). In the adjuvant setting after radical UTUC surgery, ADC-immunotherapy strategy has also advanced. The single-arm phase II study NCT05917158 evaluated adjuvant RC48 plus toripalimab in patients with HER2-overexpressing (2+/3+) UTUC who had not received neoadjuvant therapy; results showed that 55.6% of patients (25/45) were cisplatin-ineligible, and preliminary follow-up indicated a 1-year DFS rate of 90.0% with manageable safety (Liu S. et al., 2025). The results showed that ADC plus immunotherapy as a novel strategy has the potential to replace cisplatin-based adjuvant chemotherapy.

For locally advanced or metastatic urothelial carcinoma (la/mUC), the continuous updates of numerous clinical trial results have significantly reconstructed the treatment regimes. ADC has been placed in a very important position. The randomized phase III EV-302/KEYNOTE-A39 study recently confirmed a sustained survival benefit of first-line EV plus pembrolizumab in previously untreated la/mUC patients, with significantly prolonged PFS (12.5 vs. 6.3 months) and OS (33.8 vs. 15.9 months) compared to chemotherapy, along with a manageable safety profile, establishing a solid evidence base for ADC plus ICI as first-line therapy (Powles TB. et al., 2025). Another randomized phase III trial, RC48-C016, evaluated first-line RC48 plus toripalimab in HER2-expressing (1+/2+/3+) la/mUC, showing an ORR of 76.1% versus 50.2% with chemotherapy, significantly superior PFS and OS, and a lower incidence of grade ≥3 TRAEs (55.1% vs. 86.9%) (Sheng et al., 2025). And for patients intolerant or ineligible for EV, platinum-based combination chemotherapy followed by avelumab maintenance has become a recommended option (van der Heijden et al., 2025). The phase III JAVELIN Bladder 100 study has demonstrated significant OS and PFS benefits from avelumab first-line maintenance in patients without progression after chemotherapy (Powles et al., 2023). Trials of other novel therapeutic regimens for advanced UC are also ongoing. The randomized phase II DISCUS study compared 3 versus 6 cycles of gemcitabine plus cisplatin or carboplatin before avelumab maintenance, revealing comparable efficacy but significantly better quality of life with three cycles, offering an optimized choice for patients poorly tolerant to platinum and suggesting the feasibility of shorter chemotherapy courses in future trials (Powles et al., 2026). And the phase II JAVELIN Bladder Medley trial explored adding the anti-Trop-2 ADC sacituzumab govitecan (SG) to avelumab maintenance in patients without progression after first-line platinum-based chemotherapy, showing prolonged PFS (11.17 vs. 3.75 months) compared to avelumab alone, though OS data were immature and grade ≥3 TRAEs were higher (69.9% vs. 0%) (Hoffman-Censits et al., 2025). However, in advanced UC patients who failed prior platinum chemotherapy and ICI, SG did not demonstrate superior OS or PFS over physician’s choice chemotherapy (Powles et al., 2025a). Other ADC agents, including BL-B01D1 (Bian et al., 2025) and SHR-A2102 (Zhong et al., 2025), are under investigation in ongoing trials and have shown promising efficacy potential.

## Challenges and prospects

The disease burden of UC is severe, necessitating enhanced targeted interventions and resource allocation in the future (Feng et al., 2025). Novel agents represented by ICIs and ADCs are hugely impacting the disease management for NMIBC, MIBC, UTUC, and la/mUC, with several new drug combinations achieving significant progress. However, integrating such numerous novel agents into the existing UC treatment system leaves the optimal drug combinations and cycles for different populations yet to be determined, and some clinical trials (like CREST (Shore et al., 2025), KEYNOTE-905/EV-303 (Vulsteke et al., 2026-02), JAVELIN Bladder Medley (Hoffman-Censits et al., 2025)) have shown notable TRAEs. Therefore, while novel agents or combination regimens confer survival benefits, future research must focus on identifying the optimal population that can tolerate and benefit most from specific combination therapies, establishing protocols for TRAE management, and further optimizing treatment regimens. These are major challenges for current and subsequent studies.

For BCa, given the high complication rates of RC and post-surgical decline in quality of life, bladder-preserving approaches have gained increasing importance (Tempo et al., 2025; Griebsch et al., 2025). The use of novel agents such as ADCs, ICIs, and drug delivery systems like TAR-200 not only improves bladder preservation rates in NMIBC but also enhances complete response rates in neoadjuvant therapy for MIBC (Vulsteke et al., 2026-02; Shen et al., 2025; Daneshmand et al., 2025; Necchi et al., 2025b). Because the bladder urothelium communicates with the external environment, this provides a natural anatomical basis for intravesical drug delivery, which offers more localized action and fewer systemic TRAEs compared to systemic therapy (Tyagi et al., 2024). Ongoing research on intravesical administration of ADC and ICI agents is underway (Chen et al., 2020; Meghani et al., 2022). Regarding the efficacy of intravesical TAR-200, the direct comparison between TAR-200 and BCG in high-risk NMIBC patients from the SunRISe-3 trial (NCT05714202) warrants attention, as it will provide key evidence for its role in NMIBC management (Necchi et al., 2023). Future large-sample clinical trials should be conducted in broader populations (especially frail patients) to explore various combinations of intravesical and intravenous administration, aiming to maximize efficacy while minimizing systemic toxicity. For UTUC patients, pre-RNU renal insufficiency is associated with poor prognosis, and the surgery itself may impair renal function and affect outcomes (Puri et al., 2025; Momota et al., 2019). Preserving functional nephrons has become a research priority to optimize long-term prognosis, and precise patient selection is a prerequisite for kidney-sparing therapy. Future trials of novel agents should target patients with absolute or relative indications for kidney-sparing treatment, leverage advances in imaging and liquid biopsy to effectively identify those who may benefit, and attempt to combine with intracavitary therapies (Wong et al., 2025-11).

Treatment is moving from nonspecific cytotoxic drugs to molecularly tailored, patient-specific and well-tolerated cancer therapies (DasGupta et al., 2023). The cell-free DNA-based liquid biopsy is increasingly used in precision tumor monitoring, and research on cell-free DNA for optimal tumor diagnostic performance is continuously ongoing (Choy and Lo, 2025). In innovative disease management, further clinical studies are needed to explore multiple avenues. Regarding the potential of liquid biopsy in UC clinical management, the MODERN trial (NCT05987241) exemplifies ongoing efforts, investigating ctDNA-guided adjuvant immunotherapy post-surgery. This trial aims to determine whether adding relatlimab to nivolumab intensifies immunotherapy and improves survival in ctDNA-positive patients, and whether nivolumab provides survival benefit in ctDNA-negative patients, thereby providing evidence for the future application of ctDNA. Furthermore, optimal dosing regimens for new drug combinations are still under investigation. For the optimal regimen duration of ADC plus ICI in advanced UC, the EV-302 study administered pembrolizumab for up to 35 cycles with continuous EV (Powles TB. et al., 2025); meanwhile, the IMPROEV trial (NCT07221942) is recruiting patients to evaluate the strategy of six cycles of EV plus pembrolizumab followed by pembrolizumab maintenance, assessing differences in efficacy and safety between sequential regimens. Additionally, innovative therapeutic combinations merit promotion, such as RC48 combined with radiotherapy showing potential efficacy and safety in adjuvant therapy for UTUC (Tao et al., 2025). And a phase I trial of personalized neoantigen vaccination plus atezolizumab, which after 39 months of follow-up, observed no recurrence in 3 of four adjuvant-treated patients and objective responses in 2 of 5 metastatic patients with measurable lesions (Saxena et al., 2025).

## Conclusion

By 2025, UC pharmacotherapy has evolved from conventional chemotherapy/BCG to an ICIs/ADCs-driven era, marked by shifts from later-line to frontline, monotherapy to combination, and systemic to locoregional-systemic synergy. Novel drug combinations have achieved breakthrough efficacy in advanced and perioperative settings, reshaping standard care, while the focus has moved from maximally tolerated to precision-oriented, function-preserving management, evidenced by bladder-/kidney-sparing strategies and biomarker-guided individualized therapy. With expanding options, optimizing safety, identifying responders, and determining optimal treatment duration and sequencing are critical for clinical translation. Future UC treatment should evolve within precision medicine toward individualized management, multimodal combination, organ function preservation, and improved quality of life.
